# Comparative Analysis of Meat Quality and Hindgut Microbiota of Cultured and Wild Bighead Carp (*Hypophthalmichthys nobilis*, Richardson 1845) from the Yangtze River Area

**DOI:** 10.3390/microorganisms13010020

**Published:** 2024-12-25

**Authors:** Abdullateef Mukhtar Muhammad, Chang Yang, Bo Liu, Cunxin Sun, Linghong Miao, Xiaochuan Zheng, Liangkun Pan, Dong Xia, Qun-Lan Zhou

**Affiliations:** 1 Wuxi Fisheries College, Nanjing Agricultural University, Wuxi 214128, China; mukmuha1989@gmail.com (A.M.M.); 2021113023@stu.njau.edu.cn (C.Y.); liub@ffrc.cn (B.L.); suncunxin@ffrc.cn (C.S.); miaolh@ffrc.cn (L.M.); 2 Key Laboratory of Aquatic Animal Nutrition and Health, Freshwater Fisheries Research Center, Chinese Academy of Fishery Science, Wuxi 214081, China; zhengxiaochuan@ffrc.cn (X.Z.); panlk@ffrc.cn (L.P.); xiad@ffrc.cn (D.X.)

**Keywords:** meat quality, e-nose, gut microbiota, bighead carp, Yangtze River

## Abstract

Wild fish are often considered more nutritionally valuable than cultured fish. This study aimed to elucidate the relationship between the gut microbiota and meat quality through the gut–muscle axis. Therefore, cultured and wild bighead carp (*Hypophthalmichthys nobilis*, Richardson 1845) from the Yangtze River were investigated to compare the differences in the meat quality and gut microbiota composition. Cultured bighead carp were collected from four intensive ponds along the Yangtze River area, while wild bighead carp were obtained from three different sites in the Yangtze River. The results showed that wild bighead carp muscle had significantly higher total saturated fatty acid (∑SFA) and total ω − 3 polyunsaturated fatty acid (∑n − 3 PUFA) content and water-holding capacity and lower lipid, histidine, and total ω − 6 polyunsaturated fatty acid (∑n − 6 PUFA) content than cultured bighead carp, while the muscle texture was not significantly different between the two groups, with the exception of the resilience. Moreover, the hindgut microbiota was analyzed using 16S rRNA high-throughput sequencing. The alpha and beta diversity differences between the cultured and wild groups were significant. The LEfSe analysis revealed *Mycobacterium*, *Longivirga,* and *Acetobacteroides* as biomarkers in cultured bighead carp, while *Clostridium_T* and other Firmicutes-associated genera were predominant in wild bighead carp. Regarding the relationship between the hindgut microbiota and meat quality, *Mycobacterium* exhibited a positive correlation with the muscle n-6 PUFA content and a negative correlation with muscle n − 3 PUFAs, while *Clostridium_T* exhibited the opposite pattern. According to the ecological network, the abundance of *Actinobacteria* could serve as a significant indicator of variations in the abundance of *Mycobacterium* and *Clostridium_T*. Consequently, differences in meat quality, particularly in the fatty acid composition, were observed between wild and cultured bighead carp. These differences may be associated with variations in the hindgut microbiota, shedding light on the gut–muscle axis.

## 1. Introduction

Fish are a critical source of high-quality protein and essential fatty acids. It is generally found that the nutritional quality of wild and cultured fish is different. The comparison of the meat quality between cultured and wild fish has garnered attention among both researchers and consumers in recent years. Meat quality plays a crucial role in consumer preferences and is essential for human health and nutrition. The concept of meat quality in fish may undergo changes over time in response to evolving personal and societal preferences, advancements in society, and the diverse entities involved in the fish supply chain [1]. It encompasses various characteristics, such as the proximate composition, amino acid composition, fatty acid composition, flavor profile, texture, water-holding capacity, and postmortem glycolysis [2], as well as the color and pH level [3]. In one study, the flesh texture, odor, and taste significantly differed between wild and farmed large yellow croaker (*Larimichthys crocea*) [4].

Consumers prioritize factors such as nutritional value, freshness, eating quality, and physical attributes like size, species, and type in determining fish meat quality [5]. While wild fish are often perceived as healthier, more nutritious, and possessing a superior taste, studies suggest that cultured fish may offer better quality under specific environmental conditions conducive to the species’ habits [6]. Studies on common carp (*Cyprinus carpio*) [7], African catfish (*Claris gariepinus*) [8], and Nile tilapia (*Oreochromis niloticus*) [8] have indicated that the meat quality of cultured fish can surpass that of wild fish when the culture environment aligns with the species’ requirements. Therefore, assessing the meat quality of both wild and cultured fish is crucial in providing consumers with accurate information on their nutritional value and flavor profiles, thereby enhancing the understanding of aquaculture and wild fish science.

The linkage between the gut microbiota and fish meat quality has not been fully understood. The fish gut harbors a diverse range of cultivable aerobic and anaerobic microbiomes, numbering between 10^4^ and 10^9^ CFU/g [9,10]. These microbiomes play crucial roles in fish growth, survival, health, nutrition, metabolism, and meat quality [11]. The introduction of the gut microbiota in the determination of fish meat quality is a relatively new aspect of research. The composition, structure, and diversity of the fish gut microbiota are influenced by various factors, such as the host genetics, gut anatomy, developmental stage, feeding habits, diet, environmental microbial community, etc. [12,13].

There has been a recent emphasis on the connection between the gut microbiota and meat quality in farm animals. The gut microbiota impacts meat quality by influencing nutrient absorption [14] and lipid metabolism [15,16], which are closely linked to meat quality [17]. The compositions and relative amounts of the main gut bacteria communities vary among species [18]. Studies on the gut microbiota–muscle axis have shown that the intestinal microbiota affects the synthesis and function of skeletal muscle [19]. *Lactobacillus plantarum* supplementation could improve skeletal muscle to increase lean mass and function (grip strength and swim time) in healthy young mice [19]. The deletion of the intestinal microbiota led to weakened muscle function in piglets [20]. A reduction in short-chain fatty acids caused by microbiota dysbiosis could weaken the growth and physiological condition of muscle tissue [20]. Therefore, regulating the gut microbiota carefully may enhance meat quality.

The bighead carp (*Hypophthalmichthys nobilis*, Richardson 1845), a prominent species in Chinese freshwater aquaculture, is naturally found in rivers and lakes, spanning from the Pearl River in Southern China to the Yellow River in Northern China. It is popular for its affordability, taste, and nutritional value. It is distinguished by its dark coloration and unique keel structure. It is a type of filter-feeding fish, usually co-cultured in the pond with other species to control eutrophication. In a study, bighead carp from a cold-water reservoir had higher levels of unsaturated fatty acids, such as eicosapentaenoic acid (EPA) and docosahexaenoic acid (DHA), and higher levels of total polyunsaturated fatty acids than those in a cultured pond [21]. Moreover, similar results were reported in a comparison of the meat quality of bighead carp from lakes and intensive ponds [22]. Natural foods and compound feeds significantly affected the intestinal bacteria of bighead carp [23]. Therefore, it is hypothesized that there are significant differences in the meat quality and hindgut microbiota between cultured and wild bighead carp. To clearly illustrate these differences, and to shed light on the relationship between meat quality and the gut microbiota, cultured and wild bighead carp from the Yangtze River area were investigated in this study.

## 2. Materials and Methods

### 2.1. Samples Collection

**Figure 1:**
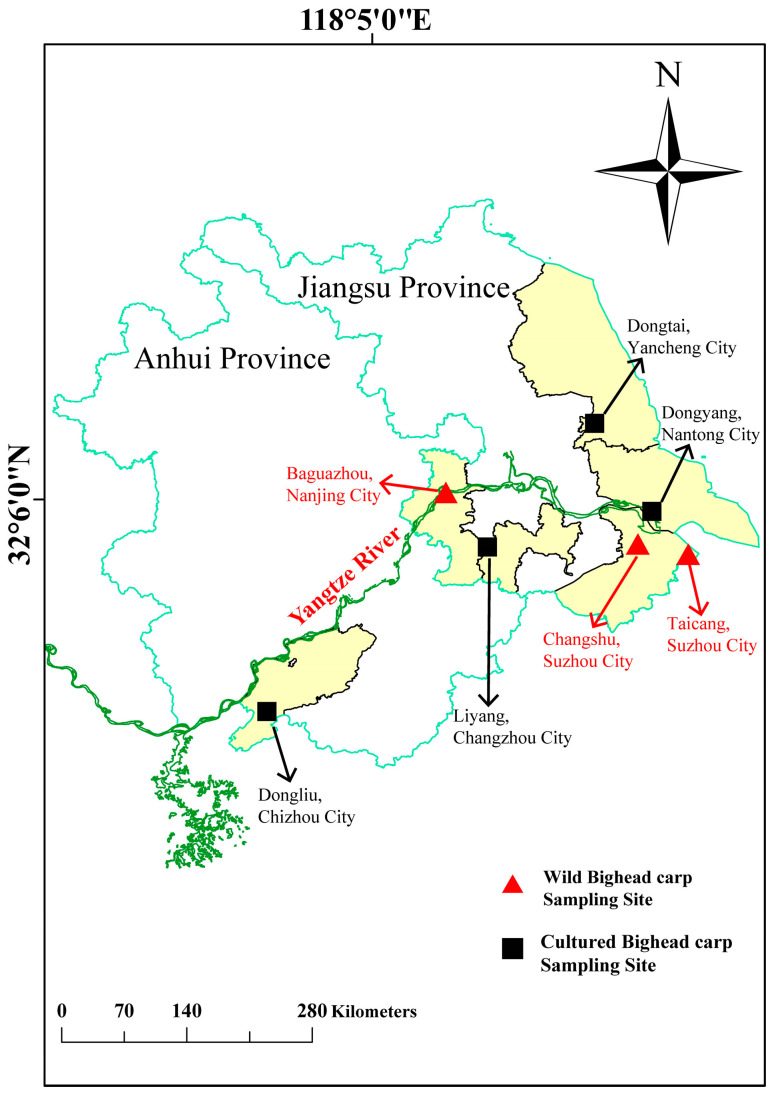
Sampling locations of cultured and wild bighead carp.

Twenty-one (21) market-size individuals were used in this study, comprising nine (9) wild bighead carp (body average length: 618 ± 24 cm) obtained from three different sites in the Yangtze River (namely Baguazhou, Changshu, and Taicang) and twelve (12) cultured bighead carp (body average length: 419 ± 20 cm) obtained from four different intensive fish farms in Dongtai County, Liyang County, and DongYang County in Jiangsu Province and Dongliu County in Anhui Province, all along the Yangtze River (E: 121°15′29′′, N: 32°52′1′′~E: 117°1′32′′, N: 29°58′0′′), during the period from June 2023 to November 2023. Figure 1 presents the sites of both the wild and cultured bighead carp. Three random sites in the middle and lower Yangtze River were selected to capture wild bighead carp. Then, cultured bighead carp were randomly collected from a nearby intensive pond. The cultured bighead carp from the intensive pond were co-cultured with grass carp (*Ctenopharyngodon idella*) and crucian carp (*Carassius auratus*). No feeding was performed for the bighead carp. Three fishes from each site were randomly chosen to collect samples. The cultured bighead carp collected at the same site came from the same hatchery with the same broodstock, while those collected in different sites were from different hatcheries with a similar genetic background; this was based on the investigation of Zhu et al. [24], using a microsatellite marker analysis.

The fish were humanely euthanized by immersion in ice-cold water to induce unconsciousness. Subsequently, dorsal muscle samples without skin were extracted from each fish through dissection using a scalpel. These samples were then vacuum-sealed, placed in separate insulated ice boxes, and transported to the laboratory. Upon arrival at the laboratory, the muscles were segmented into six parts for different analyses: proximate composition, amino acid profile, fatty acid profile, texture and water-holding capacity analysis, electronic nose analysis, and postmortem glycolysis. These samples were stored at −80 °C until use.

Gut samples were collected from the hindgut of each fish sample following the methods described by Luo et al. [25]. These samples were then placed in sterile screw-cap tubes containing sterile phosphate-buffered saline and glycerol and stored at −80 °C until they were processed further.

### 2.2. Muscle Body Composition Analysis

#### 2.2.1. Proximate Composition Analysis

The fish muscles’ moisture, crude protein, lipid, and ash content were determined using the AOAC [26] standard procedures. In brief, the muscle samples were dried to a consistent weight at 105 °C in order to measure the moisture. Using an automatic Kjeldahl machine, the crude protein method was applied to quantify the amount of protein. The crude protein was estimated by multiplying the total nitrogen amount by a factor of 6.25. The Soxhlet method of ether extraction was used to test crude lipids. By burning the ash for 12 h at 550 °C in an electric furnace until a white ash was obtained, the amount of ash was measured.

#### 2.2.2. Amino Acid Composition Analysis

The amino acid composition was determined according to the method of Chen and Zhang [27], with sight modification. A 10 mL ampoule containing 0.1 g of the material was filled with 5 mL of a 6 mol/L HCl solution. The ampoule was filled with nitrogen and sealed, and the sample was hydrolyzed at 130 °C for 7 h. The volume of hydrolysate was adjusted to 100 mL with distilled water after hydrolysis. One milliliter of hydrolysate was lyophilized and dissolved with 1 mL 0.02 mol/L HCl solution. An automatic amino acid analyzer (L-8900, Hitachi, Tokyo, Japan) was used to determine the amino acid composition after the solution had been filtered via a 0.22 µm microporous membrane.

#### 2.2.3. Fatty Acid Composition Analysis

The fatty acid composition analysis was conducted following the methods outlined by Gladyshev [28]. Lipids were extracted from the fish muscle using mechanical homogenization with a chloroform/methanol mixture (2:1, *v*/*v*) three times. The extracted lipids were then hydrolyzed under reflux at 90 °C for 10 min in a methanolic sodium hydroxide solution with a concentration of 8 mg/mL. Next, the mixture was combined with an excess methanolic solution of 3% sulfuric acid and refluxed at 90 °C for 10 min to produce fatty acid methyl esters (FAMEs). The resulting mixture was washed twice with portions of NaCl saturated solution, and the FAMEs were extracted using a portion of hexane. The FAMEs were analyzed using a gas chromatograph equipped with a mass spectrometer detector (GC-2030, Shimadzu, Kyoto, Japan) and a 30 m long, 0.25 mm internal diameter capillary HP-FFAP column (Shimadzu, Kyoto, Japan).

### 2.3. Determination of Muscle Physicochemical Properties

#### 2.3.1. Muscle Texture Determination

Texture parameters such as hardness, springiness, cohesiveness, gumminess, chewiness, resilience, and shear force were assessed using a TA-XT plusC texture analyzer (Stable Micro Systems, Godalming, Surrey, UK). The analyzer was equipped with a flat-bottomed cylindrical probe P/36R and had a force capacity and load cell of 500 N each. For the texture profile analysis (TPA) measurements, we used 2 cm × 2 cm × 2 cm pieces from the dorsal white muscle of each fish. The probe was pressed down into the sample at a constant speed of 1 mm/s until it reached 60% of the sample’s height. Each TPA test involved compressing the sample once.

#### 2.3.2. Determination of Muscle Water-Holding Capacity (WHC)

To mitigate the impact of distinct muscle regions on the WHC, 1.20 g of muscle was taken from the same site for each analysis. Using the techniques of Caimi et al. [29], the drip loss and cooking loss were used to assess the WHC of the samples. In short, to calculate the drip loss, the ipsilateral fresh back muscle was weighed, placed in a centrifuge tube (the bottom of which was cushioned with an appropriate amount of absorbent paper to absorb the water on the back muscle), and centrifuged for 20 min at 4 °C and 4000 rpm. After centrifuging the muscle sample, the water was removed using absorbent paper and then weighed.

Muscle drip loss = 100 × (W_0_ − W_t_)/W_0_

W_0_ is the mass of the muscle before centrifugation; W_t_ is the mass of the muscle after centrifugation.

Fresh back muscle was collected, weighed, dried with absorbent paper to eliminate water and blood stains, placed in a centrifuge tube, sealed in a bag, and cooked at 70 °C for 15 min in order to calculate the cooking loss. After allowing the dried muscle to cool, it was dried using absorbent paper and weighed. Using the following formula, the muscle cooking loss was determined:

Muscle cooking loss (CL) = 100 × (W_0_ − W_t_)/W_0_

W_0_ is the mass of the muscle before cooking; W_t_ is the mass of the cooked muscle.

#### 2.3.3. Determination of Muscle pH and Postmortem Glycolysis-Related Indices

The muscle pH was measured using a pHS-25 pH meter (Shanghai INESA Instrument Co. Ltd., Shanghai, China). In order to determine the muscle postmortem glycolysis, about 0.15 g of muscle was homogenized in 9 times the volume (*w*/*v*) of ice-cold normal saline at 50 Hz for 20 s, 4 times. The supernatants were then collected after the samples were centrifuged for 10 min at 4 °C and 4000 rpm to obtain 10% muscle homogenates. The levels of muscle lactic acid (LA) and glucose, as well as the activity of pyruvate kinase (PK), lactate dehydrogenase (LDH), and phosphofructokinase (PFK), were measured using commercial kits (Nanjing Jiancheng Bioengineering Institute, Nanjing, China) in accordance with the manufacturer’s instructions.

#### 2.3.4. Electronic Nose Analysis

The fish meat was crushed; then, a 2.00 ± 0.05 g sample was weighed and placed in a 20 mL injection bottle and sealed. Samples were detected by a Fast GC Alpha MOS Heracles Electronic Nose (Heracles II, Alpha MOS, Toulouse, France). The chromatographic columns were the MXT-5 and MXT-1701. The column length was 10 m and the column diameter was 180 μm. Among them, the MXT-5 was a weakly polar column and the MXT-1701 was a moderately polar column. The chromatographic peak information was processed by Alphasoft. The headspace bottle was placed at 60 °C and the sample was incubated for 300 s at 500 rpm; then, the extract was injected into the electronic nose system at a speed of 125 μL/s with a headspace injection needle at 200 °C and a 30 mL/min carrier gas flow rate (with the help of 10 kPa hydrogen N7.0 carrier gas) and maintained for 15 s. The capture system was maintained at 60 °C for 18 s to adsorb, pre-concentrate, and accumulate volatiles in the top air. The flow rate of the syringe and trap was 30 mL/min and 10 mL/min, respectively. Volatile compounds were introduced into the column at an initial pressure of 80 kPa. The initial column temperature was 50 °C, holding for 2 s and then rising to 80 °C at 1 °C/s; finally, it was increased to 250 °C at 2 °C/ s, holding for 60 s. During the total acquisition time of 177 s and the digitization cycle of 0.01 s, the signal was obtained using an FID detector (260 °C).

### 2.4. Sequencing and Bioinformatics Analysis

Nine cultured bighead carp and nine wild bighead carp were chosen to analyze the hindgut microbiota. The genomic DNA of the bacteria was then extracted from each sample using the QIAamp DNA Stool Mini Kit (Qiagen, Hilden, Germany). The quality, integrity, and concentration of each DNA sample were assessed using the established methods of Liu et al. [30]. Libraries were constructed and sequenced using the Illumina MiSeq sequencing platform. The V3–V4 hypervariable region of the *16S rRNA* gene was amplified by PCR on each sample using the universal primers 343F (5′-TACGGRAGGCAGCAG-3′) and 798R (5′-AGGGTATCTAATCCT-3′). A unique 6 bp error-correcting barcode was inserted into one of the reverse primers for each sample. Sequencing was performed by Personalbio Technology Co. Ltd. (Shanghai, China).

The raw data underwent initial filtration using the open-source software framework Quantitative Insights into Microbial Ecology (QIIME2) (version 2022.11) quality filters [31,32]. Subsequently, the sequences were further trimmed using the DADA2 (version 1.1) method [33], which involved steps such as primer removal, mass filtering, denoising, splicing, and chimerism. Amplicon sequence variants (ASVs) were obtained by de-duplicating the sequences and clustering them at 100% similarity after applying DADA2 quality control. Additionally, a representative sequence was chosen for each ASV, and taxonomic data were assigned to these representative sequences using the classify-sklearn algorithm [34]. The alignment of the sequences was performed using Greengenes2 (version 2022.10).

For the alpha diversity analysis, we utilized the QIIME2 program to calculate various indices, including Chao1, Good’s coverage, Faith’s pd, and the observed species richness estimator. Principal coordinate analysis (PCoA) using the unweighted pair-group method with arithmetic means (UPGMA) was employed to visualize changes in the microbial community structure across different samples. In order to ascertain the differential species between groups, i.e., biomarkers, the LDA effect size (LEfSe) analysis was adopted using the nonparametric factorial Kruskal–Wallis (KW) sum-rank test. According to the Metabolic Pathways From all Domains of Life (MetaCyc, version 28.5) database (https://metacyc.org/ (accessed on 1 Octorber 2019)), the abundance of two levels of metabolic pathway information was observed.

### 2.5. Correlation Analysis

The potential connection between the gut microbiota at the genus level and meat quality indices with significant changes was analyzed by Spearman’s correlation analysis. A heatmap was created to show the correlations and statistical differences using the R package (version 4.4.2). Co-occurrence networks were constructed by Spearman’s correlation analysis based on the gut microbiota in the wild and cultured bighead carp, focusing on the genera whose abundances were in the top 15 in the two groups. A *p*-value of 0.05 and a correlation coefficient of 0.6 were set. The visual networks were created using Gephi (version 0.9.7).

### 2.6. Statistical Analysis

Data were submitted to the software SPSS (v. 23.0, SPSS Inc., Michigan Avenue, Chicago, IL, USA) for analysis, and the results were expressed as the mean ± standard error. An independent-sample T-test was used to evaluate differences in the nutrient composition, amino acids, fatty acids, and texture between cultured and wild bighead carp, and they were compared at *p* < 0.05. Spearman’s correlation analysis was used to analyze the relationship between the gut microbiota and amino acids, fatty acids, texture, and water-holding capacity. GraphPad Prism (version 9.0) was used to carry out the principal component analysis (PCA) of the e-nose data based on multivariate analysis and also to draw the graphs. Data on the gut microbiota were analyzed on the Persona Genomics cloud platform, available at https://www.genescloud.cn/ (accessed on 26 January 2024).

## 3. Results

### 3.1. Muscle Proximate Composition and Physicochemical Properties of Cultured and Wild Bighead Carp from the Yangtze River Area

The muscle proximate compositions of the cultured and wild bighead carp are presented in Table 1. No obvious difference was found in the moisture, crude protein, or ash between the cultured and wild bighead carp (*p* > 0.05). However, the crude lipids were significantly higher in the cultured bighead carp than the wild population (*p* < 0.05).

As shown in Table 1, the results revealed that the hardness, springiness, cohesiveness, gumminess, chewiness, and shear force did not differ significantly between the cultured and wild bighead carp groups (*p* > 0.05). The drip loss was significantly lower in the wild bighead carp (*p* < 0.05), while the cooking loss between the cultured and wild bighead carp did not differ significantly (*p* > 0.05).

Furthermore, both the lactic acid and glucose content were not significantly different in the cultured and wild bighead carp groups (*p* > 0.05). However, the cultured bighead carp had a higher glucose concentration than the wild bighead carp. There was a significant difference (*p* < 0.05) in the activity of LDH between the cultured and wild bighead carp muscles. Likewise, the activity of PK and the PFK showed no obvious difference between the two bighead carp groups (*p* > 0.05).

### 3.2. Amino Acid Composition of Cultured and Wild Bighead Carp from the Yangtze River Area

Table 2 shows the amino acid compositions of the muscle samples of wild and cultured bighead carp. Seventeen amino acids were identified, including nine EAAs and eight NEEAs. The amino acids responsible for taste, namely aspartic acid, glutamine, serine, glycine, threonine, alanine, proline, and tyrosine, were identified. There was no significant difference in the composition of taste amino acids, total essential amino acids (∑EAA), total non-essential amino acids (∑NEAA), and total amino acids (∑TAA) in cultured and wild bighead carp (*p* > 0.05). However, the histidine in the cultured group was significantly higher than that in the wild group, while methionine showed the opposite result (*p* < 0.05).

### 3.3. Fatty Acid Compositions of Cultured and Wild Bighead Carp from the Yangtze River Area

The fatty acid profiles of the bighead carp muscle from the cultured and wild environments are presented in Table 3. Thirteen fatty acids were detected in the cultured bighead carp muscle, whereas eleven fatty acids were detected in the wild bighead carp muscle. The content of total saturated fatty acids (∑SFA) and total ω − 3 polyunsaturated fatty acids (∑n − 3 PUFA) and the ratio of ∑n − 3 PUFA/∑n − 6 PUFA in the cultured bighead carp muscle were significantly lower than those in the wild bighead carp muscle (*p* < 0.05). The significant difference in the muscle total ω − 6 polyunsaturated fatty acid (∑n − 6 PUFA) content between the cultured and wild bighead carp groups was contrary to the above trend (*p* < 0.05). The total monounsaturated fatty acid (∑MUFA) and total polyunsaturated fatty acid (∑PUFA) content were not significantly different between the cultured and wild bighead carp muscles (*p* > 0.05).

PUFAs were the main fatty acids in the bighead carp muscle (approximately 46 mg/g), followed by MUFAs (approximately 23 mg/g) and SFAs (approximately 30 mg/g). The SFAs in bighead carp muscle were mainly composed of palmitic acid (C16:0, approximately 20 mg/g) and stearic acid (C18:0, approximately 8 mg/g). The content of palmitic acid and stearic acid in the muscles of wild bighead carp was significantly higher than that in cultured bighead carp (*p* < 0.05).

The major MUFA in the bighead carp muscle was oleic acid (C18:1n9c, approximately 15 mg/g). The oleic acid content in the cultured bighead carp muscle was significantly higher than that in the wild bighead carp muscle (*p* < 0.05). The PUFAs in the bighead carp muscle predominantly consisted of docosahexaenoic acid (DHA) (C22:6n3), eicosapentaenoic acid (EPA) (C20:5n3), arachidonic acid (ARA) (C20:4n6), and linoleic acid (C18:2n6c). The EPA and DHA content were higher in the wild bighead carp muscle and were significantly different from those in the cultured bighead carp (*p* < 0.05). Likewise, linoleic acid was higher in the cultured bighead carp, with a significant difference compared to the wild bighead carp (*p* < 0.05).

### 3.4. E-Nose Analysis of Cultured and Wild Bighead Carp from the Yangtze River Area

**Figure 2:**
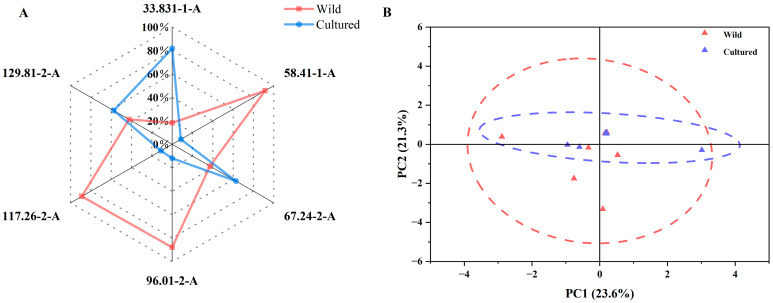
E-nose analysis of flavor of cultured and wild bighead carp from the Yangtze River area. (**A**) Radar chart. (**B**) Principal component analysis (cultured *n* = 12, wild *n* = 9).

The capacity of the e-nose to assign the bighead carp flesh samples to either the wild or culture group based on the flavor was tested using radar and PCA plots. As shown in Figure 2A, the cultured bighead carp muscles presented higher responding values for the 33.831-1-A, 67.24-2-A, and 129.81-2-A sensors, while the wild bighead carp had higher responding values for the 58.41-1-A, 96.01-2-A, and 117.26-2-A sensors. The plot of the PCA analysis in Figure 2B shows that the characteristic values representing the muscle flavor of wild bighead carp had a wider distribution range and a larger ellipse area, while the above indices in cultured bighead carp showed the opposite. Furthermore, the ellipses of the two groups had a large overlap area.

### 3.5. Gut Microbial Analysis

#### 3.5.1. Gut Microbial Composition

**Figure 3:**
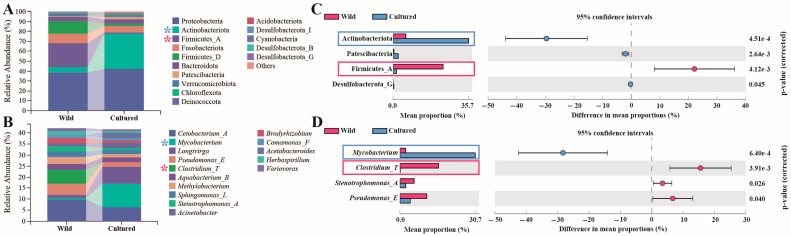
Hindgut microbiota composition in bighead carp between cultured and wild environments at the phylum and genus levels (*n* = 9). (**A**) Stacked column chart representing the phylum composition with top 15 abundance. (**B**) Stacked column chart representing the genus composition with top 15 abundance. (**C**) Bar chart of phyla with significant differences between two groups. (**D**) Bar chart of genera with significant differences between two groups. * and rectangles in red indicate high abundance in wild bighead carp, while * and rectangles in blue indicate high abundance in cultured bighead carp.

At the phylum level, the hindgut of the wild bighead carp was mainly dominated by Preteobacteria, Firmicutes, and Fusobacteriota, in this order, while that of the cultured bighead carp was dominated by Preteobacteria, Actinobacteriota, Fusobacteriota, and Bacteriota, in this order (Figure 3A). Among the phyla with the top 15 levels of abundance, those of Actinobacteriota, Patescibacteria, and Desulfobacterota_G in the cultured fish were significantly higher than those in the wild group, while the Firmicutes_A abundance in the cultured group was significantly lower than that in the wild group (*p* < 0.05).

At the genus level, *Cetobacterium*, *Clostridium_T*, and *Pseudomonas*, in this order, were the most abundant microbiota in the guts of the wild bighead carp, while *Mycobacterium*, *Longivirga*, and *Cetobacterium*, in this order, dominated the guts of the cultured bighead carp (Figure 3B). Among the genera with the top 15 abundance levels, only that of *Mycobacterium* in the cultured group was significantly higher than that in the wild group, while the abundances of *Clostridium_T*, *Pseudomonas_E*, and *Stenotrophomonas_A* in the cultured group were significantly lower than those in the wild group (*p* < 0.05).

#### 3.5.2. Richness and Diversity Analysis

**Figure 4:**
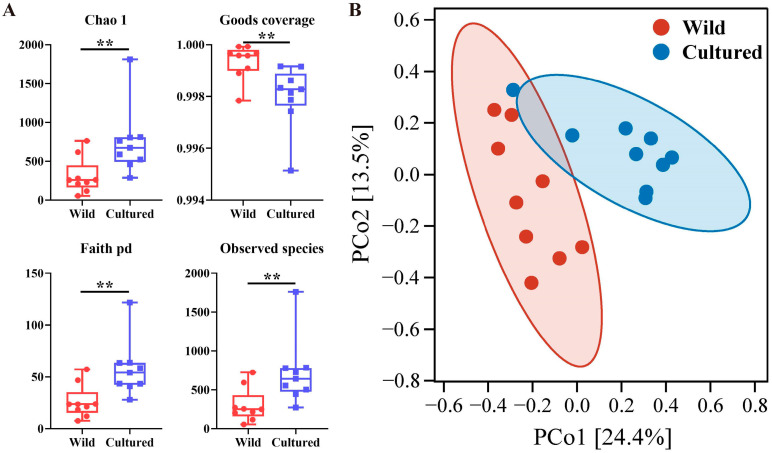
Disparities in alpha and beta diversity of bighead carp hindgut microbiota between cultured and wild environments (*n* = 9). (**A**) Calculation of Chao1, Good’s coverage, Faith’s pd, Shannon, and the observed species for bighead carp with varying dietary habits in cultured and wild environments; (**B**) PCoA plot illustrating the gut microbial structure of bighead carp growing in different environments. ** *p* < 0.01.

As presented in Figure 4A, the values of Chao1, Faith’s phylogenetic diversity, and the observed species in the cultured bighead carp were significantly higher than those in the wild bighead carp (*p* < 0.05). The Good’s coverage of the cultured bighead carp was significantly lower than that of the wild bighead carp (*p* < 0.05). According to the PCoA plot (Figure 4B), the clear separation of the hindgut microbial communities between the cultured and wild bighead carp was observed.

#### 3.5.3. Bacterial Signatures in Wild and Cultured Bighead Carp

**Figure 5:**
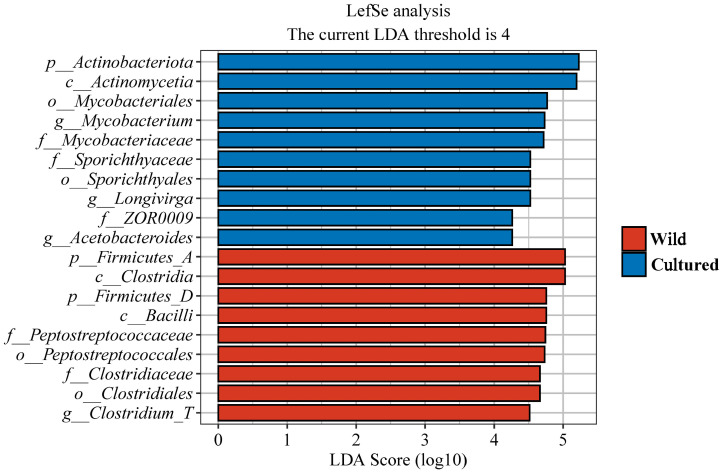
Linear discriminant effect size (LEfSe) analysis of gut microbiota composition of bighead carp between cultured and wild environments (LDA > 4) (*n* = 9).

A linear discriminant (LDA) effect size (LEfSe) analysis of the hindgut microbiota of the cultured and wild bighead carp from the Yangtze River was conducted, and the logarithmic LDA score was set = 4. Figure 5 shows that one phylum (Actinobacteriota), one class (Actinomycetia), two orders (Mycobacteriales, Sporichthyales), three families (Mycobacteriaceae, Sporichthyaceae, ZOR0009), and three genera (*Mycobacterium*, *Longivirga*, *Acetobacteroides*) were biomarkers in the hindgut microbiota of cultured bighead carp. In the hindgut microbiota of wild bighead carp, two phyla (Firmicutes_A, Firmicutes_D), two classes (Clostridia, Bacilli), two orders (Peptostreptococcales, Clostridiales), two families (Peptostreptococcaceae, Clostridiaceae), and 1 genus (*Clostridium_T*) were observed as biomarkers.

#### 3.5.4. Functional Enrichment Analysis of Gut Microbiota Between Wild and Cultured Bighead Carp

**Figure 6:**
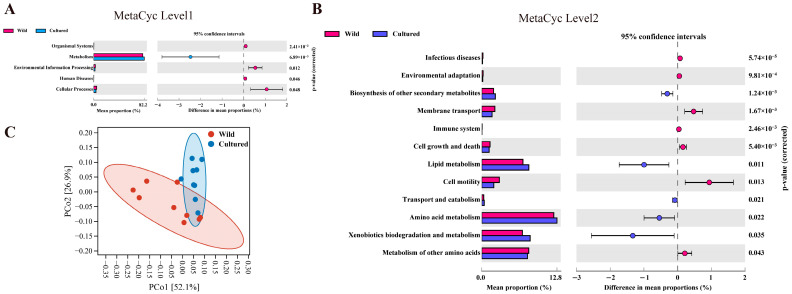
Metabolic functional profiles of bighead carp gut microbiota between cultured and wild environments (*n* = 9). (**A**,**B**) Metabolic functional profiles of bighead carp gut microbiota between cultured and wild environments at MetaCyc level 1 and level 2; (**C**) PCoA plot for functional units of bighead carp gut microbiota between cultured and wild environments.

At MetaCyc level 1, the gut microorganisms were enriched in key functional categories such as metabolism, environmental information processing, and cellular processing (Figure 6A). At MetaCyc level 2, notable distinctions were observed in the abundance of diet-related functional categories between farmed and wild cold-water fishes, including lipid metabolism, amino acid metabolism, and so on (*p* < 0.05) (Figure 6B). Furthermore, the PCoA plot indicated that the functional categories of cultured and wild bighead carp could be distinctly discerned (Figure 6C).

### 3.6. Correlation Analysis

**Figure 7:**
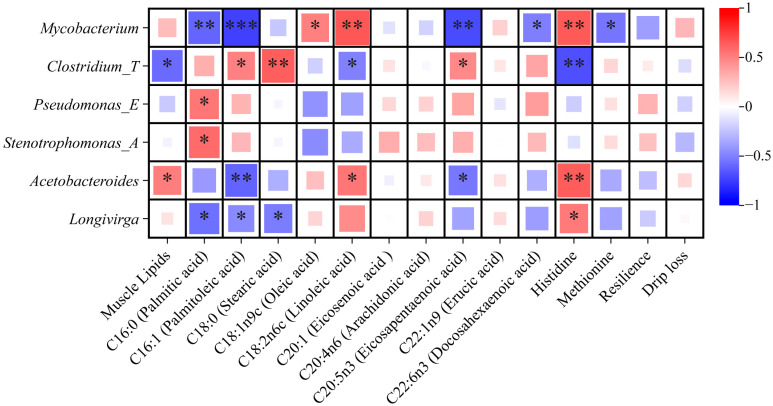
The correlations of the key gut bacteria and fish meat quality components (amino acids, fatty acids, texture, and water-holding capacity) with significant changes (*n* = 18). * *p* < 0.05, ** *p* < 0.01, *** *p <* 0.001.

The relationship between the hindgut microbiota at the genus level and the fish meat quality components (amino acids, fatty acids, texture, and water-holding capacity) with significant changes was explored using Spearman’s correlation analysis (Figure 7). The *Mycobacterium* abundance was positively correlated with C18:1n9c, C18:2n6c, and histidine, whereas it was negatively correlated with C16:0, C16:1, C20:5n3, C22:6n3, and methionine (*p* < 0.05). For *Clostridium_T*, an increase in its abundance could significantly improve the C16:1, C18:0, and C20:5n3 content, as well as significantly reducing the lipid, C18:2n6, and histidine content in the muscle (*p* < 0.05). The C16:0 content in the muscle showed significant and positive correlations with the abundance of *Pseudomonas_E* and *Stenotrophomonas_A* (*p* < 0.05). The *Acetobacteroides* and *Longivirga* abundances exhibited significant and negative correlations with the C16:1 content and significant and positive correlations with the histidine content (*p* < 0.05). In addition, the *Acetobacteroides* abundance was significantly and positively correlated with the content of muscle lipids and C18:2n6c, whereas it was significantly and negatively correlated with the content of C20:5n3 (*p* < 0.05). There were significant and negative correlations between the *Longivirga* abundance and the C16:0 and C18:0 content in the muscle (*p* < 0.05).

**Figure 8:**
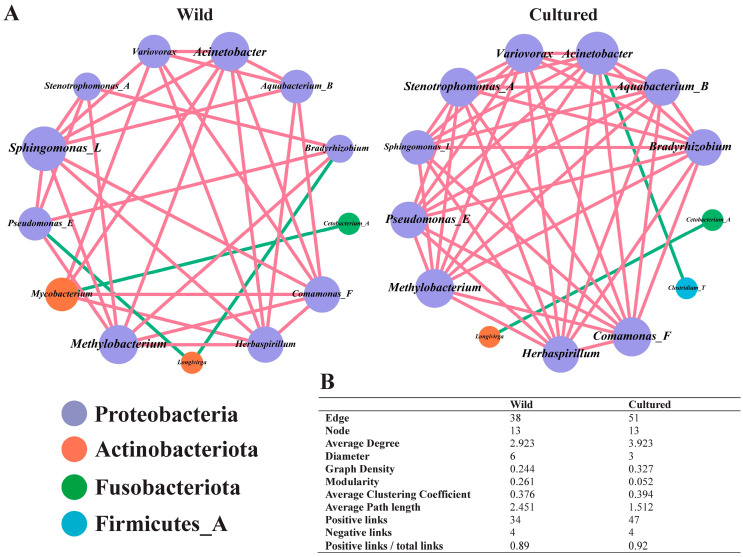
Correlation-based network analysis of hindgut microbiota community (*n* = 18). (**A**) Interspecies interaction network of bighead carp hindgut microbiota in cultured and wild bighead carp. Each node represents a genus. Node colors indicate genus affiliated with different major phyla. The green edge indicates a negative interaction between two individual nodes, whereas the red edge indicates a positive interaction. (**B**) Topological properties of hindgut microbiota community network.

As presented in Figure 8, the network relationship diagram of the genera with the top 15 levels of abundance in the cultured group consisted of 51 nodes and 13 edges, and the network diagram in the wild group consisted of 38 nodes and 13 edges. Compared with the wild group, the number of “edges”, average degree, and average clustering coefficient in the network diagram of the cultured group were higher. Interestingly, in the wild bighead carp gut, the *Mycobacterium* abundance was positively correlated with the abundance of *Herbaspirillum*, *Variovorax*, *Comamonas_F*, and *Acinetobacter*, whereas it was negatively correlated with the abundance of *Cetobacterium_A*. In the cultured bighead carp gut, the *Clostridium_T* abundance exhibited a negative correlation with the *Acinetobacter* abundance. The results showed that a change in *Acinetobacter* abundance was an important factor affecting the colonization of *Mycobacterium* and *Clostridium_T*, which are the two key genera in the guts of cultured and wild bighead carp.

## 4. Discussion

### 4.1. Muscle Proximate Composition and Physicochemical Properties of Cultured and Wild Bighead Carp from the Yangtze River Area

In this study, cultured bighead carp exhibited similar moisture, crude protein, and ash compared to wild bighead carp. This is in line with reports on natural lakes, reservoirs, and intensive ponds [21,22]. The crude protein of bighead carp in ponds and natural lakes or in intensive ponds and high-nutrition natural reservoirs are similar. Similar findings have been reported for Yellow River carp (*Cyprinus carpio haematopterus*) [2] and black rockfish (*Sebastes schlegelii*) [35]. Conversely, wild bighead carp showed lower lipid content, possibly due to their increased energy requirements for activities like feeding, reproduction, and migration in a challenging environment. In contrast, cultured bighead carp have limited activity, resulting in lower energy consumption and higher lipid accumulation. Similar trends have been observed in Yellow River carp [2].

The muscle texture is affected by the muscle collagen content, myofiber density, muscle water-holding capacity, etc. [36]. In this study, both the cultured and wild bighead carp showed similar levels of muscle hardness, springiness, cohesiveness, gumminess, chewiness, and shear force. This similarity in the muscle texture between cultured and wild bighead carp may be related to the similar muscle moisture and crude protein levels in these fish [1,36]. The reliance was significantly higher and the drip loss was lower in wild bighead carp than in cultured carp. This is consistent with findings for loach (*Misgurnus anguillicaudatus*) [37] and *Monopterus albus* [38]. The high reliance and low drip loss may be related to the high myofiber density [37,38] and the exercise exhibited by these fish [36]. The active and prolonged swimming exercise undertaken by wild bighead carp for activities like feeding, migration, and reproduction in their natural habitats may have enhanced the myofiber density and in turn enhanced the binding force of water molecules within their muscles, thereby improving their reliance and decreasing the drip loss. More attention should be paid to the muscle collagen content and myofiber density in future studies.

During the process of glycolysis, lactate dehydrogenase (LDH) is crucial in the production of lactic acid [39]. In the present study, the muscle LDH levels were significantly elevated, while the concentration of lactic acid was improved slightly in the cultured bighead carp. The plankton and natural foods for cultured bighead carp were more abundant than in wild bighead carp, which proves that cultured bighead carp consume more energy than wild fish, which promotes the accumulation of glycogen in the muscles of cultured bighead carp. After bighead carp die, more LDH is needed to participate in the conversion of glycogen to lactic acid, leading to higher postmortem LDH activity in the muscle of cultured bighead carp than in wild bighead carp. A similar phenomenon was found in a study of grass carp [40].

### 4.2. Muscle Amino Acid, Fatty Acid Composition and E-Nose Flavor of Cultured and Wild Bighead Carp from the Yangtze River Area

In addition to serving as protein building blocks, amino acids like aspartic acid, glutamic acid, serine, glycine, threonine, alanine, tyrosine, and phenylalanine directly influence fish flavor development. In this study, glutamic acid, aspartic acid, lysine, and leucine were found to be the most abundant amino acids. Arginine, leucine, and lysine constituted over half of the EAAs in both cultured and wild bighead carp. These amino acids are also predominant in Yellow River carp [2] and tilapia [41]. The TAA level of cultured bighead carp exceeded that of wild bighead carp, aligning with findings in Yellow River carp [2] and Dojo loaches (*Misgurnus anguillicaudatus*) [42]. However, the EAA levels were similar in both the cultured and wild bighead carp groups. The discrepancies in the individual EAA levels, such as histidine and methionine, between the two groups may stem from EAA imbalances in their respective foods. The level of histidine in the muscle of cultured bighead carp was significantly higher than that in wild bighead carp in this study. This is similar to a comparison between intensive pond cultures and reservoirs, where the authors observed higher histidine levels in cultured bighead carp than in the fish in the reservoirs [22]. This might be related to the commercial feed. Although the cultured bighead carp were not fed in this study, the farmers still fed other co-cultured carp with commercial feed. Thus, the bighead carp might have been fed with residues or zooplankton and been affected by the commercial feed indirectly. It was also reported that the histidine levels of bighead carp in a common culture pond were lower than those in Poyang Lake, which was also attributed to the plankton composition [21].

The fatty acid composition and content are pivotal in assessing fish muscle quality and flavor [43]. In the current study, wild bighead carp exhibited higher levels of SFAs compared to cultured bighead carp, with C16:0 and C18:0 being the primary SFAs. Similar results were reported in studies of black rockfish [35] and seabass/seabream (*Dicentrarchus labrax*) [44]. Usually, SFAs such as C14:0 and C16:0 increase the risk of cardiovascular disease as they are positively related to low-density lipoprotein (LDL) and cholesterol. However, it was found that dietary SFAs raise the plasma lipid content only when the diet lacks n-3PUFAs. Dietary SFAs are beneficial or neutral when they are taken with the recommended n-3PUFA levels together and they are part of a healthy diet [45,46]. C18:1n9c was the major MUFA in all bighead carp muscles and was significantly higher in cultured bighead carp, which is consistent with reports on crayfish (*Procambarus clarkii*) [47] and seabass [48]. The EPA and DHA (n − 3 PUFAs) levels were higher in the wild bighead carp in this study, suggesting that the muscle of the wild bighead carp has higher nutritional value compared to cultured bighead carp, as EPA and DHA from fish or fish oil are beneficial in lowering the risk of fatal cardiac events and atrial fibrillation [49]. This indicates that wild bighead carp are more beneficial for human health. A similar phenomenon was observed in the Mongolian redfin (*Chanodichthys mongolicus*) [50] and black rockfish [35]. The n-6 PUFAs, particularly C18:2n6c, were more prevalent in the cultured group. Moreover, the wild bighead carp muscles exhibited a higher Σn − 3/Σn − 6 PUFA ratio (2.61 mg/g) compared to their cultured counterparts (0.76 mg/g), suggesting a richer n-3 PUFA environment in the wild habitat (Yangtze River). A similar finding was noted in Rio Grande silvery minnows (*Hybognathus amarus*) [51] and seabass [48]. Overall, the fatty acid components of wild bighead carp demonstrated superior nutritional quality. The differences in the fatty acid composition between the cultured and wild bighead carp in this study may be attributed to variations in food composition, food intake, feeding practices, and environmental factors like the temperature, season, and location [52].

The flavor as detected by an e-nose plays a crucial role in evaluating the acceptability and palatability of food. As the radar map indicated, both the cultured and wild bighead carp had three higher sensor values (cultured: 33.831-1-A, 67.24-2-A, and 129.81-2-A; wild: 58.41-1-A, 96.01-2-A, and 117.26-2-A). This shows that the muscles of fish living in different environments have their own characteristic flavors. At the same time, it can be seen from the PCA plot that the wild bighead carp group had a larger ellipse than the cultured bighead carp group, indicating that that the flavor of the cultured bighead carp muscles was not as rich as that of the wild bighead carp. An important reason for this discrepancy could be the different fatty acid compositions of the muscles between cultured and wild bighead carp. These results also reveal that the muscle flavor can be used to distinguish between cultured and wild bighead carp.

### 4.3. Gut Microbiota of Cultured and Wild Bighead Carp from the Yangtze River Area

The host habitat is the main factor that determines the gut microbiome of fish [53]. Proteobacteria were the most dominant taxa in this study, which was consistent with other studies of bighead carp [25,54]. *Cetobacterium* was the most abundant genus observed in the hindguts of bighead carp. As the genus *Cetobacterium* has been speculated to have a role in the synthesis of vitamin B12 in the fish gut, and its prevalence mainly contributes to the production of vitamin B12 in humans [55,56], wild bighead carp, with high levels of *Cetobacterium*, would be more nutritionally valuable for human health. However, in this study, vitamin B12 in the muscle and gut was not investigated; further studies on this topic could be considered.

Meanwhile, the abundances of *Mycobacterium* and *Clostridium_T* differed significantly between the two groups. From the LEfSe analysis, *Mycobacterium* and *Clostridium_T* were identified as biomarkers for cultured and wild bighead carp, respectively. This is similar to a report comparing the gut microbiomes of bighead carp under different rearing strategies [57] and a report comparing reservoirs and lakes [58]. The intensive pond experienced more eutrophication than the Yangtze River due to feeding. Therefore, the environmental microbiota in the Yangtze River was significantly different from that in the intensive pond. Formulated feeds cause the *Cetobacterium* and *Mycobacterium* abundance to increase compared with the natural live food [57]. The wild bighead carp in the Yangtze River only have natural live food, while cultured bighead carp might be affected by formula feeds for grass carp and crucian carp, either directly or indirectly. The high abundance of *Mycobacterium* observed in cultured bighead carp may be attributed to its widespread distribution in aquatic environments, particularly in the water and sediments of intensive pond systems. Additional investigations on more individuals with different rearing or living conditions are needed to clarify the microbiota differences and microorganism biomarkers.

Moreover, *Mycobacterium* is related to a chronic disease, mycobacteriosis, which may not present symptoms but causes losses due to poor growth, lower survival, and poor productive responses [59,60]. Some *Clostridium* spp., such as *Clostridium butyricum,* are reported to be nutritional contributors to the host, especially regarding fatty acids and vitamins [61]. Meanwhile, other species are opportunistic pathogens, such as *Clostridium perfringens*, which is a common anaerobic pathogen found in freshwater fish in Kashmiri Himalayan lakes [62]. It is difficult to accurately locate species-level bacteria based on 16S rRNA high-throughput sequencing. More advanced methods, such as whole-genome sequencing, should be considered when analyzing gut microbes in further studies.

Not only the species composition but the diversity of the gut microbiota exhibited notable distinctions. The alpha diversity index revealed higher species diversity and numbers in the guts of the cultured group compared to the wild group, while distinct characteristics of the gut microbial communities in the fish living in different environments were found in the PCoA analysis in this study. However, in a previous investigation of cold-water fish, the alpha diversity index of the cultured group was not as high as that of the wild group [63]. This phenomenon may arise from variations in species, water microorganisms, and food sources.

The disparity in the microbiota community structure may lead to different functions, as confirmed by the PCoA plot regarding the functional units between the two groups. Specifically, in the present research, an observable upward trend in the metabolic functional category abundance of wild and cultured bighead carp was noted. For example, there were pronounced differences in the pathways linked to amino acid and lipid metabolism within the gut microbiota of bighead carp. Interestingly, the abundance of the lipid metabolism pathway in the cultured group exhibited a remarkable increasing trend compared with the wild group, consistent with the results regarding the muscle content and a study of cold-water fish [63]. The adaptive responses of bighead carp to distinct habitats and food sources may contribute to this diversification and differentiation in their functional pathways [63].

### 4.4. Correlation Analysis to Identify Gut Microbiota Effects on Muscle Quality and to Distinguish Wild and Cultured Bighead Carp

The ‘gut–muscle axis’ concept (i.e., the impact of the gut microbiota and its interaction with the host’s muscle metabolism and function) was first introduced regarding the cross-talk between the gut microbiota and skeletal muscle health. Such research mainly focused on the changes in aging skeletal muscle [19,64]. Then, it was proven in germ-free piglets that the gut microbiota was closely related to the development and function of skeletal muscle [20]. Moreover, *Lachnoclostridium* was proven to be associated with the drip loss rate and abdominal fat rate of Beijing-You broilers [65]. A similar result was reported in tilapia (*Oreochromis niloticus*), where faba bean (*Vicia faba* L.) altered the microbiota to change the lipopolysaccharide pathways, thus altering the meat texture [66]. The gut microbiota can directly or indirectly affect the muscle quality. Therefore, the correlations between important genera and meat quality components with significant changes were explored in this study.

Consistent with the results of the functional analysis, the key genera in the cultured group, such as *Mycobacterium*, *Acetobacteroides*, and *Longivirga*, exhibited more correlations with lipid and amino acid metabolic indicators. For instance, these genera were all positively correlated with the muscle histidine content. Histidine is closely related to the growth and antioxidant statuses of aquatic animals and is utilized for energy production during adverse conditions [67]. In terms of the muscle lipid composition, the key genera in the cultured group were positively correlated with n-6 PUFAs (LA) and negatively correlated with some beneficial n − 3 PUFAs (EPA and DHA). Moreover, an increase in *Acetobacteroides* abundance may give rise to muscle lipid deposition. On the contrary, the gut microbiota biomarker in the wild group, *Clostridium_T*, showed a negative correlation with muscle lipids and positive correlations with EPA and DHA. It has been proven that some *Clostridium* species, such as *Clostridium butyricum,* produce short-chain fatty acids during the fermentation of carbohydrates and are closely related to the growth performance, immune response, and disease resistance, as well as the modulation of the gut commensal microbiota and metabolic disorders, in aquatic animals [10]. Therefore, it is possible that the gut microbiota could be reshaped to increase the n-3 PUFA levels in carp via probiotics regulation in aquaculture. Then, cheap carp could be exploited for their benefits for human health. More research work, such as fecal microbiota transplantation, is needed to confirm the positive correlation between *Clostridium* and PUFAs.

Through constructing the gut microbiota ecological network, the cooperation and competition among different genera whose abundances were in the top 15 could be represented visually, which helped to investigate the mechanisms by which key genera regulate muscle quality. In the context of this research, the gut microbiota complexity in the cultured bighead carp was higher than that in the wild bighead carp, based on the edge number and positivity rates [68]. In the ecological network of the two groups, the abundance of *Acinetobacter* was a significant indicator regarding the variations in the abundances of two key genera, *Mycobacterium* and *Clostridium_T*. It may be possible to regulate the colonization of *Mycobacterium* and *Clostridium_T* in the guts of bighead carp living in different environments by adjusting the abundance of *Acinetobacter*. Studies of more individuals are needed in the future to confirm this finding.

## 5. Conclusions

The present study demonstrates that the meat quality and hindgut microbiota vary between cultured and wild bighead carp. Wild bighead carp possesses higher EPA and DHA levels and thus higher nutritional value compared to cultured bighead carp. Different hindgut biomarkers, such as *Mycobacterium* and *Clostridium_T*, were identified in this study. Meanwhile, this study established a significant relationship between the hindgut microbiota and muscle fatty acids. *Acinetobacter* was a significant indicator of the abundances of two key genera, *Mycobacterium* and *Clostridium_T*, based on the ecological network. The results of this study shed light on the relationship between meat quality and the gut microbiota and expand and enrich the research on the “gut–muscle axis”, although they need further confirmation with more individuals.

## Figures and Tables

**Table 1:** Muscle proximate composition and physicochemical properties of cultured and wild bighead carp from the Yangtze River area.

	Culture Method		Significance
Index	Cultured	Wild	(*p*-Value)
Moisture (%)	74.95 ± 0.71	75.91 ± 0.55	ns
Crude protein (%)	21.11 ± 0.41	20.45 ± 0.28	ns
Lipid (%)	2.12 ± 0.64	1.82 ± 0.06	*
Ash (%)	1.64 ± 0.07	1.58 ± 0.07	ns
Hardness (g)	1852.80 ± 211.49	1761.15 ± 128.88	ns
Springiness	0.385 ± 0.02	0.383 ±0.02	ns
Cohesiveness	0.402 ± 0.03	0.440 ± 0.034	ns
Gumminess	761.56 ± 112.27	780.54 ± 92.71	ns
Chewiness	304.87 ± 53.17	308.29 ± 49.66	ns
Resilience	0.16 ± 0.01	0.19 ± 0.01	*
Shear force (N)	1.26 ± 0.13	1.33 ± 0.22	ns
Drip loss (%)	15.54 ± 1.58	12.21 ± 0.91	*
Cooking loss (%)	13.84 ± 1.17	13.71 ± 2.23	ns
pH	5.71 ± 0.05	5.84 ± 0.07	ns
Lactic acid (µmol/mgrot)	49.53 ± 4.19	46.89 ± 3.88	ns
Glucose (mmol/grot)	4.06 ± 0.45	3.36 ± 0.46	ns
LDH (U/gprot)	7114.04 ± 86.12	6692.01 ± 179.41	*
PK (U/gprot)	169.94 ± 20.99	140.34 ± 11.56	ns
PFK (U/mgrot)	4.95 ± 0.49	4.06 ± 0.45	ns

Note: The data are represented as mean ± S.E. (cultured *n* = 12, wild *n* = 9); * means significant difference (*p* < 0.05); ns, non-significant; LDH, lactate dehydrogenase; PK, pyruvate kinase; PFK, phosphofructokinase.

**Table 2:** Amino acid compositions of cultured and wild bighead carp from the Yangtze River area.

Amino Acid (g/100 g)	Cultured	Wild	Significance (*p*-Value)
*Essential amino acids*			
Histidine	0.56 ± 0.02	0.45 ± 0.01	*
Threonine	0.80 ± 0.02	0.79 ± 0.11	ns
Arginine	1.18 ± 0.02	1.18 ± 0.02	ns
Valine	0.88 ± 0.01	0.91 ± 0.02	ns
Methionine	0.52 ± 0.01	0.60 ± 0.01	*
Phenylalanine	0.83 ± 0.01	0.83 ± 0.01	ns
Isoleucine	0.81 ± 0.02	0.86 ± 0.01	ns
Leucine	1.59 ± 0.02	1.59 ± 0.03	ns
Lysine	1.87 ± 0.03	1.85 ± 0.04	ns
*Non-essential amino acids*			
Aspartic acid	2.35 ± 0.04	2.28 ± 0.05	ns
Glutamic acid	3.41 ± 0.06	3.35 ± 0.06	ns
Serine	0.82 ± 0.01	0.79 ± 0.01	ns
Glycine	0.89 ± 0.01	0.88 ± 0.03	ns
Alanine	1.11 ± 0.01	1.11 ± 0.02	ns
Tyrosine	0.59 ± 0.01	0.58 ± 0.01	ns
Cystine-s	0.09 ± 0.00	0.07 ± 0.00	ns
Proline	0.72 ± 0.01	0.72 ± 0.04	ns
Taste AA	10.79 ± 1.15	10.60 ± 0.75	ns
∑EAA	9.04 ± 0.19	9.06 ± 0.19	ns
∑NEAA	9.98 ± 0.15	9.78 ± 0.21	ns
∑TAA	19.02 ± 0.34	18.84 ± 0.41	ns

Note: The data are represented as mean ± S.E. (cultured *n* = 12, wild *n* = 9). Taste AA: taste amino acids, including aspartic acid, glutamic acid, glycine, threonine, alanine, tyrosine, and phenylalanine. EAA: essential amino acid, NEAA: non-essential amino acid, TAA: total amino acids; * means significant difference (*p* < 0.05); ns: non-significant.

**Table 3:** Fatty acid compositions of cultured and wild bighead carp from the Yangtze River area.

Fatty Acid (%)	Cultured	Wild	Significance (*p*-Value)
C14:0	2.09 ± 0.42	2.59 ± 0.33	ns
C16:0	18.54 ± 0.39	21.05 ± 0.44	*
C16:1	2.88 ± 0.35	4.92 ± 0.45	*
C18:0	7.01 ± 0.32	8.75 ± 0.34	*
C18:1n9c	16.92 ± 1.14	12.31 ± 1.11	*
C18:2n6c	15.47 ± 1.34	3.01 ± 0.27	*
C20:1	3.85 ± 0.60	4.23 ± 0.65	*
C20:2	1.23 ± 0.10	-	ns
C20:3n6	1.82 ± 0.18	-	ns
C20:4n6 (ARA)	9.22 ± 0.49	9.44 ± 0.54	*
C20:5n3 (EPA)	7.57 ± 1.00	14.52 ± 1.19	*
C22:1n9	0.62 ± 0.09	0.50 ± 0.05	*
C22:6n3 (DHA)	12.77 ± 0.83	18.02 ± 1.21	*
∑SFA	27.64 ± 0.60	32.39 ± 0.91	*
∑MUFA	24.27 ± 2.00	21.96 ± 2.92	ns
∑PUFA	48.08 ± 1.78	45.00 ± 3.41	ns
∑n − 3 PUFA	20.34 ± 2.55	32.54 ± 3.67	*
∑n − 6 PUFA	26.51 ± 1.85	12.45 ± 0.53	*
∑n − 3 PUFA/n − 6 PUFA	0.76 ± 0.13	2.61 ± 0.35	*

Note: The data are represented as mean ± S.E. (cultured *n* = 12, wild *n* = 9); * means significant difference (*p* < 0.05); - means not detected; ns: non-significant. ARA, arachidonic acid; EPA, eicosapentaenoic acid; DHA, docosahexaenoic acid; SFA, saturated fatty acid; MUFA, monounsaturated fatty acid; PUFA, polyunsaturated fatty acid; n − 3 PUFA, omega-3 polyunsaturated fatty acid; n − 6 PUFA, omega-6 polyunsaturated fatty acid.

## Data Availability

The data, except the sequence data, are available from the corresponding author on reasonable request. The sequence data that support the findings of this study have been deposited in the NCBI with the primary accession code PRJNA1180720.
